# Steam-cooking rapidly destroys and reverses onion-induced antiplatelet activity

**DOI:** 10.1186/1475-2891-11-76

**Published:** 2012-09-20

**Authors:** Emilie A Hansen, John D Folts, Irwin L Goldman

**Affiliations:** 1 Department of Health Professions, Metropolitan State University of Denver, Denver, CO, USA; 2 Department of Medicine, Cardiology Section, H6/379 CSC, University of Wisconsin-Madison, Madison, WI, 53702, USA; 3 Department of Horticulture, University of Wisconsin-Madison, 1575 Linden Drive, Madison, WI 53706, USA

**Keywords:** Onion, Platelets, Antiplatelet, Cooking, Polyphenols

## Abstract

**Background:**

Foods in the diet that can aid in the prevention of diseases are of major interest. Onions are key ingredients in many cuisines around the world and moreover, onion demand has trended higher over the past three decades. An important pharmacological aspect of onion is the ability to inhibit platelet aggregation. Raw onions inhibit platelet aggregation; however, when onions are boiled or heated, antiplatelet activity may be abolished.

**Methods:**

Onion quarters were steamed for 0, 1, 3, 6, 10, and 15 min. The in vitro antiplatelet activity of a yellow hybrid storage onion was examined at these times on the blood of 12 human subjects using in vitro whole blood aggregometry.

**Results:**

Contrary to findings reported for boiling, antiplatelet activity was destroyed between 3 and 6 min of steaming, and at 10 min of steaming, cooked onions stimulated platelet activity. Extracts from cooked onion had the potential to reverse the inhibitory effect on blood platelets by 25%. Responses were consistent across all donors. Total polyphenolic concentration and soluble solids were not affected by steaming time.

**Conclusions:**

The potential value of cooked onion preparations may result in destruction or reversal of antiplatelet activity, without affecting the polyphenolic concentration.

## Background

Onions (*Allium cepa* L) have a world-wide importance in culinary practice, given that they add unique flavors to fresh and cooked food [1]. It is well known that not every onion tastes the same; flavor ranges from very mild to extremely pungent when in raw form [1]. There are many factors influencing flavor in onions. The genetic background of an onion partially determines its capacity for flavor however the growing environment plays an important role in the modification of flavor composition [2]. High sulfate availability, high growing temperatures, and dry conditions generate more pungent onions for any given cultivar [3].

A unique class of organosulfur compounds forms the primary determinants of onion flavor [3]. Three odorless, non-volatile flavor precursors collectively referred to as S-alk(en)yl cysteine sulfoxides (ACSOs) are stored in the cytoplasm of intact onions: *S*-propyl cysteine sulfoxide (PCSO), *S*-methyl cysteine sulfoxide (MCSO), and *S*-(E)-1-propenyl cysteine sulfoxide (1-PeCSO) [4,5]. The intensity or pungency can be predicted by the differences in the concentrations of these precursors. Alliinase, an enzyme compartmentalized in the cell’s vacuole, is released upon tissue disruption and reacts with these non-volatile precursors. ACSOs are cleaved by alliinase to sulfenic acids, ammonia, and pyruvate [3]. The sulfenic acids are chemically unstable and will combine with another of the same species to give a range of sulfur compounds. Sulfenic acids condense to form thiosulfinates (TSs). Thiosulfinates are also unstable and thus undergo dissociation and rearrangement to form primary and secondary volatiles, along with secondary nonvolatile compounds [3].

The balance between pungency and levels of sugars determines the perception of sweetness in an onion. Because of their low pungency, mild onions are primarily eaten raw and are added to foods including, salads, sandwiches, dips, dressings and cold side dishes. Full-flavored pungent onions possess a higher concentration of sulfur-based compounds that overpower the perception of sweetness [1]. Consequently, pungent onions are used in cooking practices to add unique base flavors and enhance the flavor of food preparations [6].

An increase awareness of health maintenance and disease prevention in the past decade has led consumers to make more informed food choices based not only on flavor, but also on enhanced nutritional value of specific foods [7]. Onions and their constituents have several remedial benefits including antiplatelet activity, antimicrobial activity, anti-inflammatory and anti-asthmatic effects [8]. Raw onions have been shown to inhibit platelets from aggregating both in vitro in human blood and ex vivo in rats [9,10].

Previous research in our laboratory and others has suggested that the onion-induced antiplatelet activity (OIAA) of raw onions and other alliums is attributed to the organosulfur compounds. In vitro responses of human platelets to four thiosulfinates have been examined singly and synergistically in whole blood [11]. Thiosulfinates (TSs) show differential antiplatelet activity in vitro, which is dependent upon the alk(en)yl substituents [11]. In addition to the thiosulfinates, onions are high in flavonoids, more specifically, the flavonols, which also have the potential to affect platelets. Onion is a major source of the 4’-substituted quercetin glucosides (quercetin-3,4-diglucoside and quercetin-4-glucoside) [12]. These two glucosides and free quercetin are reported to constitute 68% of the total polyphenolic content and over 85% of the flavonoid content in the onion bulb [13]. In addition to quercetin, onion contains kaempferol, kaempferol conjugates, rutin, iso-rhamnetin and iso-rhamnetin conjugates. Many flavonoids, including quercetin, were found to inhibit blood platelet aggregation in vitro and in vivo [14].

The extent of the contribution of organosulfur compounds and flavonoids to OIAA is unknown. Furthermore, the extent of degradation or loss of these two classes of compounds during cooking is unknown. Extracts from boiled Welsh onion (*Allium fistulosum*) juice showed in vitro platelet-stimulating effects [15]. If boiled onions regularly induce platelet activity in humans, this may be a cause for concern among consumers. Onions, especially pungent types possessing high levels of organosulfur compounds, are typically cooked prior to eating, rather than eaten raw, thereby potentially amplifying this concern. Following the in vitro study, Chen et al., fed rats raw or boiled (30 min) Welsh onion juice for 4 weeks and showed that raw juice consumption significantly inhibited platelet function while boiled juice was completely ineffective and did not have stimulating effects [9]. Cavagnaro et al. showed that boiling onions for 3 min did not affect OIAA compared to raw onion, but did find a suppressive effect on OIAA of >6 min of boiling. Pro-aggregatory effects were noted after more than 20 min of boiling [16].

Onions are domestically prepared by many different cooking techniques, such as frying, sautéing, blanching, microwaving, baking, grilling and steaming. The implication of cooking technique on OIAA has not been studied extensively. In the present study, we chose steaming as a method of onion preparation. Steaming has been shown to minimize flavonoid and other nutrient losses in vegetables [17,18]. In addition, steaming prevents impurities, leaving salts, acids, alkaline and heavy metals to remain in the water portion without affecting the vegetable. Our main objective was to examine the effects of cooking on OIAA across multiple human blood donors. Our secondary objectives were to observe the extent of total polyphenol loss and change in soluble solids following cooking. We hypothesized that onions would lose antiplatelet activity and total polyphenols gradually over steaming time and subjects would respond similarly.

## Methods

### Chemicals

Saline, sodium citrate, Folin-Ciocalteu reagent, gallic acid (1 mg/mL), and sodium carbonate were obtained from Sigma-Aldrich (St. Louis, MO). Collagen was obtained from Chrono-log Corp. (Havertown, PA).

### Onions

500 mature onion bulbs from a hybrid storage onion trial grown at Hancock, WI were harvested at random and stored at 5°C until use.

### Preparation of onion samples

On the day of the blood draw, after removing the dry outer scales, onions were randomly selected and quartered. Onion quarters (16 quarters/treatment) each (32.7 ± 11.1 g) fresh weight were (i) steamed for 0 min (raw) (ii) steamed for 1 min (iii) steamed for 3 min (iv) steamed for 6 min (v) steamed for 10 min or (vi) steamed for 15 min. General descriptions of onions are given in Table 1. A domestic stovetop (General Electric) was used. Tap water (500 mL) was added to a stainless steel saucepan (1 L) and boiled before and during steaming. An expandable stainless steel basket was set in the base of the saucepan and eight onion quarters were evenly spaced on the steamer. It was important to prevent water from contacting onions, thereby eliminating the possibility of direct leaching. Immediately after steaming, core temperatures of onion quarters were taken and quarters were juiced with a commercial juicer (Table 1). The resulting juice was filtered through cheesecloth into five 1.5 ml centrifuge tubes. The juice was incubated at 22°C for 30 min to allow ACSO lysis by alliinase and then centrifuged twice at 4000 × g for 10 min at 4°C to remove any extra pulp. The resulting supernatant was stored on ice immediately before use.

**Table 1:** Description of onion extracts (0 min, 1 min, 3 min, 6 min, 10 min, 15 min steamed)

**Time (min)**	**Fresh wt (g)**	**Temp (°C)**	**Texture**
0 min (raw)	30.56 ± 3.89	17.22 ± 2.47	0.0
1 min	31.76 ± 4.66	43.755 ± 9.17	0.5
3 min	30.79 ± 3.96	66.03 ± 5.52	1.5
6 min	31.87 ± 4.58	76.06 ± 7.33	2.5
10 min	35.73 ± 4.39	84.90 ± 4.27	4.0
15 min	35.02 ± 3.72	90.42 ± 2.34	5.0

Weights are fresh weights reflective of quartered onions before cooking. Temperature is reflective of the core temperature of quartered onions immediately following cooking. Texture is scored on a 0–5 scale (0 = hard and 5 = soft).

### Subjects

Twelve healthy subjects (six males and six females) from the University of Wisconsin volunteered for the study (University of Wisconsin Health Sciences Human Subject Committee protocol #M-2004-1242). The average age was 38 year (range 22–65 year), average weight was 68.14 kg (range 52–94 kg), and average BMI was 22.6 kg/m^2^ (range 20.4-25.3 kg/m^2^). Volunteers agreed to abstain from non-steroidal anti-inflammatory drugs, aspirin, tea, coffee, wine, and beer for 5–7 d prior to blood draw. A diet high in fruits and vegetables was also discouraged 2–4 d prior to blood draw. The Institutional Review Board at the University of Wisconsin approved the protocol and subjects signed informed consent prior to participation.

### In vitro incubations

Blood was drawn by venipuncture from twelve donors through a 19-gauge butterfly needle into a syringe containing sodium citrate (1 volume 3.8% sodium citrate to 9 volumes blood) and mixed gently. The blood was then mixed with an equal volume of 9 g/L preservative-free buffered saline and maintained at 22°C during the experiment and used within 3 h of venipuncture.

In vitro platelet aggregation was measured in a two-channel whole-blood aggregometer (Chrono-log Model 5, Havertown, PA). Graphical output was recorded on a chart recorder (Chrono-log, Havertown, PA). Onion juice was tested for platelet inhibition at 100 mL juice/L blood. This concentration was determined in pilot studies to be the most effective and likely most realistic concentration (data not shown). All onion extracts were repeated four times in the blood of each subject. Blood was drawn from each donor twice on two separate days. Platelet aggregation was induced by collagen (2 mg/L). After the addition of the collagen agonist, the platelet response was recorded over 7 min and the maximal peak height at 7 min was used in the final analysis. Results are expressed as the percentage of platelet aggregation inhibition by onion juice.

### Total polyphenolics

The total polyphenolic content of each of the six onion groups were assayed using the Folin-Ciocalteu reagent [19,20]. The Folin-Ciocalteu reagent was prepared by diluting a stock solution with distilled water (1:10, v/v). Test tubes were prepared with gallic acid standards (10, 20, 30, 80, 150, 200, 250 μL) or a sample of onion juice. 5 mL of diluted reagent and 4 mL of Na_2_CO_3_ (75 g/L) was added to each of the test tubes. The tubes were inverted twice and kept at room temperature for 2 h. Absorbance at 675 nm was recorded for the onion juices and gallic acid standards. The results are given in milligrams per milliliter of gallic acid equivalent (GAE).

### Soluble solids

Total dissolved solids content of each of the six onion groups were determined by absorbance refractometery, as described by Mann & Hoyle (1945). Soluble solids were measured three times for each extract using 10 μL of onion juice. Data are expressed as the percent total dissolved solids averaged for the three replicates for each onion extract at each cooking time point.

## Results and discussion

### In vitro incubations

**Figure 1:**
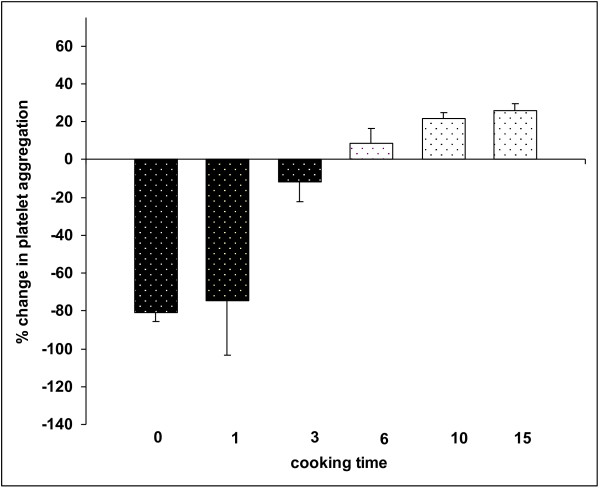
**Effect of cooked onion extracts on human platelet aggregation in vitro.** Negative values are platelet inhibitory, the magnitude of the value reflects the amount of inhibition relative to a parallel control. Positive values are platelet stimulatory reflecting the percentage increase in platelet aggregation above a collagen-induced control cause by the addition of the extract.

Analysis of variance revealed that the interaction of subject x cooking time was not significant for OIAA (P = 0.40). Thus, the main effect of cooking time averaged over subject was examined (Figure 1). Human platelets in whole blood responded significantly different to cooking times (P < 0.0001). The baseline for each subject served as a control for each subject. Percent changes from baseline at 0 min (raw), 1 min, 3 min, 6 min, 10 min and 15 min of steaming were -81.0 ± 14.4, -74.7 ± 14.4, -11.7 ± 5.3, +8.4 ± 3.9, +21.9 ± 1.5, +25.7 ± 1.8, respectively. Raw juice significantly inhibited the collagen-induced platelet aggregation response (P < 0.0001). Steaming onions for 1 min resulted in a significant inhibition of platelet activity (P < 0.0001), however, steaming onions for 3 min did not result in a significant inhibition of platelet activity (P = 0.39). Steaming onions for 10 min and 15 min significantly promoted collagen-induced platelet aggregation (P = 0.02 and P = 0.01, respectively). Platelet aggregation was increased 22% and 25% by steaming onions for 10 and 15 min., respectively.

### Total polyphenolics

**Figure 2:**
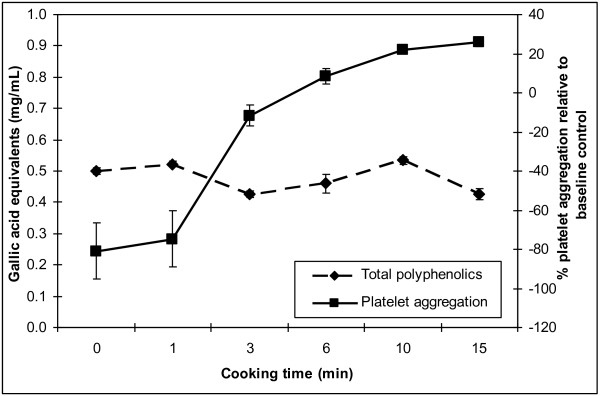
**Mean polyphenolic concentration (by Folin-Ciocalteu’s procedure) of six onion extracts.** The concentration was expressed as mg/ml of gallic acid equivalents shown on the left side. No significant change in GAE was detected (P = 0.147). Platelet aggregation was significantly different among extracts.

Total polyphenolics did not change over cooking time (P = 0.1470). The total phenolic content and percent change in aggregation over cooking time are shown in Figure 2. The mean values for 0 min (raw), 1 min, 3 min, 6 min, 10 min and 15 min were 0.50 ± 0.02, 0.52 ± 0.02, 0.43 ± 0.02, 0.46 ± 0.06, 0.54 ± 0.03, and 0.48 ± 0.04 gallic acid equivalents (mg/mL), respectively.

### Soluble solids

**Figure 3:**
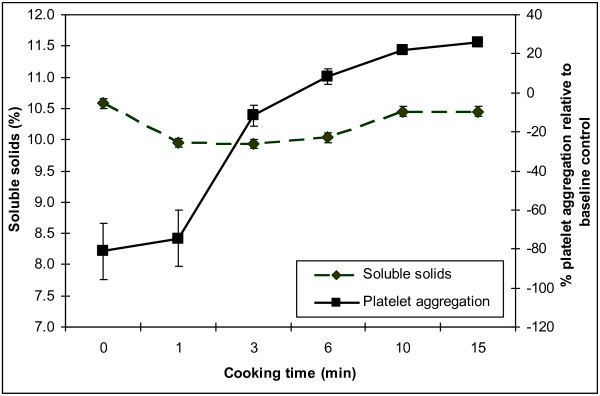
**Mean percent soluble solids of the six onion extracts.** No significant change in soluble solids over time was detected (P = 0.2091). Platelet aggregation was significantly different.

Percent total dissolved solids did not change over cooking time (P = 0.2091). Soluble solids content and percent change in aggregation over cooking time are shown in Figure 3. The mean values for 0 min (raw), 1 min, 3 min, 6 min, 10 min, and 15 min were 10.58 ± 0.0, 9.95 ± 0.1, 9.93 ± 0.4, 10.03 ± 0.2, 10.45 ± 0.2 and 10.45 ± 0.1, respectively.

Antiplatelet activity is not specific to the edible Alliums. Whole foods and beverages such as fish oil [21], flavanol rich chocolate [22], pomegranate juice [23], purple grape juice [23,24], and red wine [25] have been shown to exhibit antiplatelet activity. Clearly, our results show raw onion juice significantly inhibits platelet activity in vitro, which is consistent with previous studies [11,26].

To our knowledge, this is the first study examining the temporal effects of steaming onion on OIAA. Other studies have looked at exclusively raw extracts compared to boiled extracts [16]. Cavagnaro et al. showed boiling for 3 min had no significant effect on OIAA, and that significant inhibition of platelet aggregation by boiled onion occurred up to 6 min. Our results showed that steaming for 3 min had large effects on OIAA, and by 6 min, significant pro-aggregatory effects were measured. The proaggregatory response increased linearly following the 6 min period. Cavagnaro et al. found the proaggregatory response to be significant at 20 min of boiling, compared to our findings that 6 min of steaming was enough to reverse the effect. Taken together, these results suggest a much more rapid destruction and reversal of OIAA from steaming.

Our results show that the longer steaming time, the greater loss of antiplatelet activity. All 12 subjects responded similarly to cooked onions. At 10 min steaming, we observed not only a complete loss of antiplatelet activity, but also a stimulatory effect on platelet aggregation compared to controls. This response could be of particular significance to those attempting to consume onions for their platelet-inhibitory properties. Previous work has shown that boiled Welsh onions (*Allium fistulosum*) caused a proaggregatory response in vitro but not in vivo in rats [9]. Even though this stimulatory effect was not found in rats, the phenomenon remains a concern. If stimulation of platelet activity occurs in vitro in humans, a possibility that this would occur in vivo remains, given the correct amount/type of cooked onion ingested and absorbed. Moreover, there is a possibility that those at risk for atherosclerosis or impaired endothelial dysfunction would be put at a greater risk, if consuming cooked onions.

Platelet aggregation plays a key role in the development of atherosclerosis, a process that begins in adolescence [27]. Atherosclerosis is a systemic proliferative and inflammatory disease of the vascular wall of arteries [28]. One of the first stages of atherosclerosis is damage to the endothelial lining of arteries [29]. When endothelial cells become damaged or dysfunctional, platelets are stimulated. Platelets are an essential element in normal hemostasis, however, when they become hyperactive, they can contribute to the progression of atherosclerosis [30]. If endothelial cells are damaged or dysfunctional and platelets are activated, platelets will aggregate and adhere to the lining of arteries [30]. Activated platelets secrete growth factors that cause luminal narrowing and intimal thickening, a major part of atherosclerosis [30]. A diet rich in natural platelet inhibitors may reduce an individual’s risk of developing or reversing the progression of atherosclerosis, however this work indicates that the method of culinary preparation of such inhibitors may be important.

Extensive work has been reported on specific beverages and foods inhibiting platelet aggregation, whereas, limited work has been reported on beverages and foods stimulating platelet aggregation. Certain fractions of grape skin have been shown to promote aggregation, however, when both stimulatory and inhibitory fractions were tested collectively, grape skin remained inhibitory as a whole [31]. In addition, epinephrine is reported to promote platelet aggregation in whole blood [32,33], however, discrepancies in reports on caffeinated beverages promoting platelet aggregation exist [34,35].

Briggs et al. [10] suggested that the thiosulfinates, other organosulfur compounds and flavonoids found in onions act synergistically to inhibit platelet activity. In this study, after examining antiplatelet activity, raw and steamed onion extracts were assayed for total phenols by Folin-Ciocalteu reagent (FCR). The Folin-Ciocalteu antioxidant capacity assay allowed an examination of polyphenols without introducing the reducing capacity of organosulfur compounds into the assay [36]. Our results demonstrated that total polyphenolics remained at similar concentrations over cooking time. Other studies have revealed diminished antioxidant activity of onion after cooking [37,38]. Agostini et al. [36] used the Trolox equivalent antioxidant capacity assay (TEAC) and found 0.223 equivalent uM Trolox in the raw onion and 0.146 equivalent uM Trolox in the boiled onion. TEAC is unspecific for polyphenols yet excellent linear correlations have been observed between TEAC and total phenolic profile using the FCR assay [36]. Differences in cooking method (steaming versus boiling) could be the source of inconsistency between the present study and previous work. Our hypothesis on why we detected no significant change in total polyphenolics is that steaming may have removed water, thus preserving the remaining polyphenols in a more concentrated form. Therefore, the constant polyphenolic concentration throughout the cooking process is plausible.

Discrepancies exist among cooking studies and quercetin content. In the present study, quercetin was not measured because it was not a primary objective of the study and total polyphenolic concentration was a greater interest than concentration of a specific polyphenol. Nevertheless, quercetin is the main polyphenolic compound found in onion; therefore many studies have measured quercetin loss during cooking. Data show that 150 mg and 300 mg quercetin-4’-O-beta-D-glucoside ingested orally in humans resulted in platelet inhibition 30 min and 120 min after ingestion [39]. Subjects given a diet containing onion slices three times a day (260-360 g/day) for one week resulted in an equivalent of 67.6-93.6 mg/day of quercetin ingestion and it’s concentration in the plasma increased from 0.04 ± 0.04 uM before the trial to 0.63 ± 0.72 uM after the trial [40]. These studies suggest that onion quercetin conjugates are accumulated in the plasma and may provoke antiplatelet effects if ingested at a high enough dose.

The effect on quercetin content is probably contributed by many factors including, cultivar differences, sample preparations, processing techniques, and assessment techniques [41]. Lombard et al. found that baking quartered onions for 15 min in a pre-heated rotisserie oven at 176°C and sautéing sliced onions in sunflower oil for 5 min on an electric skillet at 93°C resulted in an increase of flavonols and quercetin conjugate contents. Twenty five percent and 7% gains of the two major glucosides (3,4’-Qdg + 4’-Qmg) were found after sautéing and baking, respectively. Additionally, the ratio of 3,4’-Qdg to 4’-Qmg conjugates decreased slightly during cooking compared to raw onions. This overall gain was attributed to concentrating the onion by removing water during cooking. Lombard et al. found an 18% decrease in the two conjugates in boiled onion compared to raw onion. Other studies examining boiling for 3–60 min have reported between 20%-75% losses of quercetin [41-46] and these losses were attributed to leaching of quercetin into the cooking water.

The present study suggests that the polyphenols, mainly quercetin conjugates, in onion juice may have little or no independent effect on platelet aggregation. In addition, onion juice may have too low a total polyphenolic content to contribute to an inhibitory effect. This may explain why platelet aggregation changed, even though no significant difference in total polyphenolic content was detected in raw and treated onions. Polyphenolics are in much higher total concentrations in other vegetables and fruits, and it is likely that the polyphenols from comparatively low polyphenolic onions are not the primary contributors to the inhibitory effect. For example, total phenolic concentration of broccoli and spinach were 80.76 ± 1.17 mg/100 g and 79.55 ± 8.39 mg/100 g [47], whereas the onions used in the present study were 47.8 ± 5.15 mg/100 g. Compared with fruits, vegetables typically have a lower total polyphenolic concentration. Cranberries, apples, red grape were analyzed and exhibited 507.0 ± 21.1 mg/100 g, 272.1 ± 6.2 mg/100 g, and 182.0 ± 2.6 mg/100 g phenolic concentrations, respectively [48]. It is possible that platelet inhibitory effects may be explained better by foods and/or beverages with a much greater antioxidant capacity than the onions used in the present study. Purple grape juice had three times the total polyphenolic concentration of orange and grapefruit juice and it had about three times greater effect on platelet inhibition [24]. More applicable to the present study, varietal differences in polyphenolic concentration exist among onions. An onion with a high value, such as, Western Yellow (104.9 ± 13.0 mg/100 g) was twice as high as the yellow onion used in this study. Polyphenols in Western Yellow could have a greater affect on antiplatelet activity more so than the onion used in the present study [49].

It is well established that organosulfur compounds in onions change dramatically with time and depend on processing method [50]. The chemistry of *Allium*-derived sulfur compounds is complex because many compounds are volatile, thermally unstable, condense or decompose to form other compounds, and under different conditions, different compounds are generated. Thiosulfinates, initial chemicals formed in freshly macerated tissues, undergo transformation and decomposition. Most flavor compounds are formed from the decomposition of thiosulfinates [50]. With regard to steaming, it has been shown that during steam distillation, oils are generated. During steam distillation, tissues are heated to 100°C and initially formed thiosulfinates are rapidly converted into their corresponding polysulfide. Block et al. found that heating pure diallyl disulfide for 10 minutes at 150°C resulted in the formation of greater than 30 sulfur components [51]. Lawson et al. found steam-distilled garlic cloves were only 35% as active in inhibiting platelets compared to aqueous garlic clove homogenates [52]. Differences in reductions in antiplatelet activity were noted between our study, which involved steaming, and other studies, which have involved boiling. It is possible that the generation of oils or polysulfides is partially responsible for this effect. Because of the unstable nature and difficulty characterizing sulfur composition in onion tissues, we were unable to provide data showing specific sulfur components found in our steamed and raw extracts. It would be of interest to determine whether platelet stimulatory fractions are present in raw onion and if their activity is countermanded by platelet inhibitory compounds in raw onion. Further analysis of sulfur components from domestically prepared onion tissues would be an important and useful measurement. More importantly, it would be useful to determine if the platelet stimulatory response is reflective of a loss of specific sulfur components, formation of different sulfur components, or is reflective of other facets related to cooking.

We sought to examine antiplatelet activity inducement of other vegetables in raw and cooked forms in a pilot study. We found that raw broccoli inhibited platelet activity in three out of the four human subjects (data not shown). Additionally, cooked broccoli did not inhibit platelet activity and became stimulatory after cooking beyond 6 min. This finding is interesting because the change from inhibitory to stimulatory may not be specific for onion, but may encompass other vegetables that are platelet inhibitors in raw form. Causes of this proaggregatory response are beyond the scope of our study, but remain of great interest to our laboratory.

Lastly, we examined soluble solids and found that they did not change significantly during the cooking process. In raw form, OIAA is correlated strongly with high pungency and high soluble solids [53]. In contrast, we found high soluble solids remained when the onion was cooked, yet OIAA changed. Since soluble solids mostly consist of carbohydrates and ASCOs make up a low percentage of total soluble solids, this finding was not surprising. However, cooking decreases pungency, therefore, OIAA and pungency may remain strongly correlated.

## Conclusions

In the present study, antiplatelet activity induced by onion extract was destroyed between 3 and 6 min of steaming. At 10 min of steaming, cooked onions stimulated platelet activity, and cooked extracts had the potential to reverse the inhibitory effect on blood platelets by 25%. Thus, possible negative health implications of eating onions in the cooked form were revealed. Because OIAA was lost and reversed with steam-cooking, consumers may want to minimize onion cooking time, choose onions with high pungency for cooking practices, and/or consume raw onions on a regular basis in order to preserve OIAA.

## Abbreviations

OIAA: Onion-induced antiplatelet activity; TS: Thiosulfinates.

## Competing interests

The authors have no specific competing financial interests relative to this study.

Author IL Goldman has a competing financial interest related to betalain pigments from table beet that are used for food coloring applications. This interest is not relevant to the present study.

## Authors’ contributions

IG, JF, and EH developed the ideas and protocols for the study. EH developed protocols that were integral to the study design and conduct. EH supervised, organized, and carried out the bioassays. EH, IG, and JF participated in the analysis and writing of the manuscript. All authors read and approved the final manuscript

## Author details

^1^Department of Health Professions, Metropolitan State University of Denver, Denver, CO, USA. ^2^Department of Medicine, Cardiology Section, H6/379 CSC, University of Wisconsin-Madison, Madison, WI 53702, USA. ^3^Department of Horticulture, University of Wisconsin-Madison, 1575 Linden Drive, Madison, WI 53706, USA.
